# IMPACT OF FRAILTY AND DEMENTIA ON REHABILITATION OUTCOMES IN OLDER ADULTS SURGICALLY TREATED FOR HIP FRACTURE

**DOI:** 10.1093/geroni/igae098.2764

**Published:** 2024-12-31

**Authors:** Giulia Caggiu, Matteo Monzio Compagnoni, Patrizia Floris, Martina Manna, Francesco De Filippi, Michela Passamonte, Paolo Mazzola

**Affiliations:** University of Milano-Bicocca, Milano, Lombardia, Italy; University of Milano-Bicocca, Milano, Lombardia, Italy; Morbegno Hospital, Morbegno, Lombardia, Italy; University of Milano-Bicocca, Milano, Lombardia, Italy; Società Italiana di Geriatria Ospedale e Territorio, Milano, Lombardia, Italy; Morbegno Hospital, Morbegno, Lombardia, Italy; University of Milano-Bicocca, Milano, Lombardia, Italy

## Abstract

We investigated the impact of frailty and dementia on rehabilitation outcomes after hip fracture (HF) surgery in an Italian Orthogeriatric Unit (OGU). We prospectively enrolled all patients aged ≥70 years admitted to the OGU at Sondrio Hospital (Italy) in 2011-2019, with HF requiring surgery. We administered a comprehensive geriatric assessment, including factors related to fracture and surgery. A frailty index (FI) based on 16 variables expressed frailty (FI=number of deficits/16), with which we obtained three categories: fit (FI< 0.15; reference), mild (0.15< FI< 0.34), moderate-to-severe (FI≥0.35). Among 818 participants (mean age 85.6 years, 83.3% females), 21.6% had pre-existing dementia. The average time-to-surgery was 1.9 days (77.6% in spinal anesthesia). Rehabilitation success, i.e. Rehabilitation Efficiency Index (REI)>0.5, was achieved by 70.2% of subjects. From multivariable logistic regression, factors impacting rehabilitation success were frailty (both categories), dementia (OR: 0.14, 95% CI: 0.10-0.21), and age ≥90 years (OR: 0.37, 0.21-0.64). The chances of rehabilitation success were lower for patients with dementia than for those without. Although there is no clear link between age and rehabilitation success, frailty has a decreasing influence as age increases. In nonagenarians, severe frailty does not limit rehabilitation success as much as in younger patients. Previous research showed that dementia potentially acts as a surrogate for frailty in predicting post-operative 1-year mortality after HF surgery. We show that dementia and frailty significantly influence rehabilitation success, thus early mobilization and initiation of physical therapy regardless of age should target non only frail subjects, but also those with dementia, possibly with specifically dedicated interventions.

